# Dietary patterns in Xinjiang, Western China and associations with metabolic syndrome: a population-based study

**DOI:** 10.1017/S1368980026102316

**Published:** 2026-04-01

**Authors:** Qian Zhao, Juan Wang, Zulipiye Xier, Na Yang, Jun Liu, Aboduhapaer Tuersunjiang, Hui Peng, Yi-Ning Yang

**Affiliations:** 1 The Cardiac and Panvascular Medicine Diagnosis and Treatment Center, People’s Hospital of Xinjiang Uygur Autonomous Region, Urumqi, China; 2 Xinjiang Key Laboratory of Cardiovascular Homeostasis and Regeneration Research, Urumqi, China; 3 Department of Cardiology, The First Affiliated Hospital of Xinjiang Medical University, China; 4 Luopu County Hanggui Town Health Center, China

**Keywords:** Dietary pattern, Metabolic syndrome, Prevalence, Western China

## Abstract

**Objective:**

To examine the association between dietary patterns and metabolic syndrome (MetS) in western China, which has not been previously reported.

**Design:**

A population based cross-sectional study design. Dietary intake was assessed using a semi-quantitative FFQ. Principal component analysis identified dietary patterns and multivariate logistic regression evaluated their associations with MetS.

**Setting:**

Population-based Cohort Study of Chronic Diseases in Xinjiang (PCCDX), conducted in 2022.

**Participants:**

A total of 3208 individuals from PCCDX (mean age: 53·1 (sd 10·8) years; 49·1 % male).

**Results:**

MetS was diagnosed in 1762 participants (54·9 %). Four distinct dietary patterns were identified, with the refined grain–animal products dietary pattern being the dominant one. After adjusting for general demographic and lifestyle factors, a higher score in the refined grain–animal product pattern was associated with an increased risk of MetS. The OR for the second, third and fourth quartiles of the dietary score were 1·07 (95 % CI: 0·860, 1·322), 1·14 (0·923, 1·413) and 1·48 (1·189, 1·853), with a statistically significant trend (*P* = 0·003). Higher dietary scores in this pattern were also associated with increased risks of elevated waist circumference, high TAG and low HDL cholesterol (*P* < 0·05). Mediation analysis showed that visceral fat percentage partially mediated the association between the refined grain–animal product dietary pattern and low HDL-cholesterol, accounting for 17·2 % of the total effect (indirect effect = 0·005, *P* = 0·006). The other three dietary patterns showed no significant associations with MetS or its components.

**Conclusions:**

This study highlights the high prevalence of MetS in western China and links a refined grains-animal products diet to poorer metabolic health, emphasising the need for region-specific dietary strategies.

Metabolic syndrome (MetS), characterised by central obesity, insulin resistance, elevated blood pressure and dyslipidaemia, represents a cluster of modifiable risk factors that substantially increase the likelihood of CVD, type 2 diabetes mellitus and other adverse health outcomes^(1,2)^. As a growing global health challenge, MetS prevalence has risen alarmingly in recent decades, driven largely by urbanisation, sedentary lifestyles and dietary transitions^(3–5)^. This trend is particularly evident in China, where prevalence more than doubled from 13·7 %^(6)^ in 2001 to 31·1 % in 2017^(7)^. Diet plays a pivotal role in both the development and prevention of MetS and its related conditions. An unbalanced diet contributes to approximately 35 % of CVD mortality, primarily by promoting excess body weight, dyslipidaemia and increased risks of diabetes and hypertension, making it one of the most critical factors in the prevention of premature CVD death^(8)^. Notably, dietary interventions have demonstrated cardiometabolic benefits even in the absence of significant weight loss or reductions in adiposity^(9)^.

Traditionally, epidemiological studies on diet and health have focused on single nutrients, a perspective that continues to shape current dietary guidelines. However, growing evidence underscores the importance of dietary patterns in managing metabolic disorders^(10–12)^, with patterns such as the Mediterranean^(9)^, DASH^(13)^ and Chinese heart-healthy diets^(14)^ showing notable associations with improved cardiometabolic outcomes^(15)^. Given that dietary patterns can vary substantially, even when nutrient intakes are broadly similar—due to cultural differences in food preferences—consideration of population-specific dietary behaviours is essential. Western China, particularly Xinjiang, remains understudied in this context. Influenced by nomadic traditions and distinct environmental, cultural and socio-economic contexts, local Uyghur diets are characterised by high intakes of animal fats and red meat, diverging markedly from typical Han Chinese diets^(16–18)^. Addressing this research gap will advance our understanding of geographically specific diet–disease relationships and inform public health strategies tailored to regional characteristics.

Our study aims to assess the prevalence of MetS and its components in western China based on data from the Population-based Cohort Study of Chronic Diseases in Xinjiang (PCCDX) and to identify regional dietary patterns in relation to MetS.

## Methods

### Study population

This study utilised baseline data from the PCCDX study to assess the prevalence of MetS and its components, as well as to identify the associations between local dietary patterns and MetS. Data collection was conducted from July to September 2022. A convenience sampling approach was used to select HangGui Town in Luopu County, Hotan Prefecture, a region predominantly inhabited by Uyghur populations. Random sampling was not feasible due to logistical and resource constraints. The potential for selection bias due to the sampling approach is acknowledged. The project team recruited in forty-two natural villages covering the whole town through health education, village medical publicity and organisation. Eligible participants were permanent residents aged 30 years or older who were able to communicate effectively, free of major physical disabilities and who provided written informed consent. The study was approved by the Ethics Committee of the People’s Hospital of Xinjiang Uygur Autonomous Region (Approval No. KY2021031904) and conducted in accordance with the principles of the Declaration of Helsinki. The study was registered as a cross-sectional investigation at the Chinese Clinical Trial Registry (ChiCTR2200056783).

### Data collection

All participants underwent assessments that included questionnaires, physical examinations and laboratory tests. Demographic characteristics, clinical history, lifestyles including drinking, smoking, diet, sleep quality and physical activity were collected using a standardised structured questionnaire, administered in the local language through one-on-one, face-to-face interviews by trained bilingual interviewers to minimise interviewer bias. Current drinking was defined as drinking at least once per week in the past year^(19)^, and current smokers were defined as smoking at least one cigarette per day or seven cigarettes per week for more than half a year^(20)^. Sleep quality and physical activity levels were assessed using the Pittsburgh sleep quality index and the International Physical Activity Questionnaire, respectively. Physical activity was categorised based on total weekly activity levels: low (< 600 MET-minutes), moderate (600–3000 MET-minutes) and high (> 3000 MET-minutes). Sedentary time was defined into three groups: < 3 h/d, 3–6 h/d and ≥ 6 h/d. BMI was categorised as follows: underweight (< 18·5 kg/m^2^), normal weight (18·5–24 kg/m^2^), overweight (24–28 kg/m^2^) and obesity (≥ 28 kg/m^2^) for Chinese^(21)^.

### Dietary evaluation

Dietary intake over the past year was assessed using a semi-quantitative FFQ. The food categories were modified based on the dietary patterns and habits of Xinjiang population, comprising a total of forty-four items across thirteen categories, including rice, flour-based food, coarse grains, desserts, livestock meat and products, poultry and products, aquatic products, eggs, vegetables, fruits, nuts, legumes and products and yogurt (online Supplementary Table 1). For each food item, participants reported their consumption frequency using five categories: ‘Daily’, ‘5–6 d a week’, ‘3–4 d a week’, ‘1–2 d a week’, ‘Monthly (≤ 3 times)’ and ‘Almost never’. The median frequency of the categorical variable was converted into a continuous variable, corresponding to an average weekly intake of 7, 5·5, 3·5, 1·5, 0·5 or 0 d, respectively. Food intake was quantified as grams per day (g/d) or milliliters per day (ml/d) using standardised dietary models and graphical aids. The final average daily intake of each food group was calculated by multiplying the reported frequency by the quantified portion size and dividing by 7 days. The questionnaire was validated through pre-testing on a small, representative sample to ensure clarity, reliability and cultural relevance. Internal consistency was assessed using Cronbach’s alpha (*α* = 0·82), indicating acceptable reliability for the questionnaire in this population.

### Laboratory tests

An overnight fasting blood sample was collected from each participant to measure blood biochemical indexes using an automatic clinical chemistry analyser (LABOSPECT 008, Hitachi, Ltd). The specific indicators measured included fasting plasma glucose (FPG), LDL-cholesterol, HDL-cholesterol, total cholesterol and TAG.

### Definition and assessment of metabolic syndrome

MetS was defined according to the Joint Interim Statement of the International Diabetes Federation Task Force on Epidemiology and Prevention (JIS)^(22)^, with modifications to the dyslipidaemia criteria based on the 2023 China Guidelines for Lipid Management^(23)^ to better reflect the Chinese population. MetS was diagnosed when three or more of the following components were present: (1) elevated waist circumference (WC): WC ≥ 85 cm (men) or ≥ 80 cm (women); (2) elevated blood pressure: systolic blood pressure (SBP) ≥ 130 mmHg (1 mmHg = 0·133 kPa) or diastolic blood pressure ≥ 85 mmHg, or receiving antihypertension treatment; (3) hypertriglyceridaemia: fasting TAG ≥ 1·7 mmol/l or lipid-lowering treatment; (4) low HDL-cholesterol: < 1·0 mmol/l or corresponding treatment and (5) elevated fasting glucose: FPG ≥ 5·6 mmol/l, antidiabetic treatment or self-reported diabetes.

### Statistical analysis

Descriptive statistics were presented as mean (sd) or median (interquartile range) for continuous variables and *n* (%) for categorical variables. The Kruskal—Wallis test was used for continuous variables, while the Pearson’s *χ* ^2^ test was used for categorical variables. Trends across groups were assessed using the Mantel–Haenszel *χ* ^2^ test. The prevalence of MetS and its components among the overall population, sex and age groups was shown using a radar map. Dietary patterns were identified through principal component analysis based on the intake levels of thirteen food groups. To optimise the factor structure, varimax rotation was applied to maximise variance while minimising the number of high-loading variables per factor. The number of dietary patterns was determined using eigenvalues greater than 1 and scree plot analysis. Food items with an absolute factor loading of ≥ 0·5 were considered primary contributors to a dietary pattern. To assess the association between dietary patterns and MetS, a multivariate logistic regression model was used. Participants were grouped into quartiles based on dietary pattern scores, with Q1 as the reference group. The model was adjusted for potential confounders, including age, gender, education level, smoking status, physical activity level, sedentary time and sleep quality (Pittsburgh sleep quality index score), which are known to influence metabolic health. BMI and WC, as MetS components, were not included to avoid collinearity. Additionally, as continuous variables, the potential nonlinear relationship of dietary patterns score with MetS and its components was estimated by a restricted cubic spline fitted in the fully adjusted logistic regression model. The restricted cubic spline models were constructed with three knots placed at the 5th, 50th and 95th percentiles of each exposure, corresponding to 3 df. Mediation analysis assessing the mediating effects of obesity indicators (BMI, WC and visceral fat) on the associations of dietary patterns with MetS and its components. The presence of a mediating effect was defined as satisfying all the following conditions having a significant indirect effect, a significant total effect and a positive proportion of the mediator effect. The analyses were conducted using the mediation package in R (version 4.2.3; R Foundation for Statistical Computing).

Sensitivity analyses using different criteria were performed to further assess the robustness of our findings. Alternative MetS definitions were applied, including the 2004-modified National Cholesterol Education Program Adult Treatment Panel III (NCEP-ATP III) criteria, the 2005 International Diabetes Federation (IDF) criteria and the 2009 JIS criteria.

All data analyses were conducted using Stata version 16.0 (Stata Corp LLC) or R software. Statistical significance was defined as a two-tailed *P* value < 0·05.

## Result

### Participants characteristics

**Figure 1.**
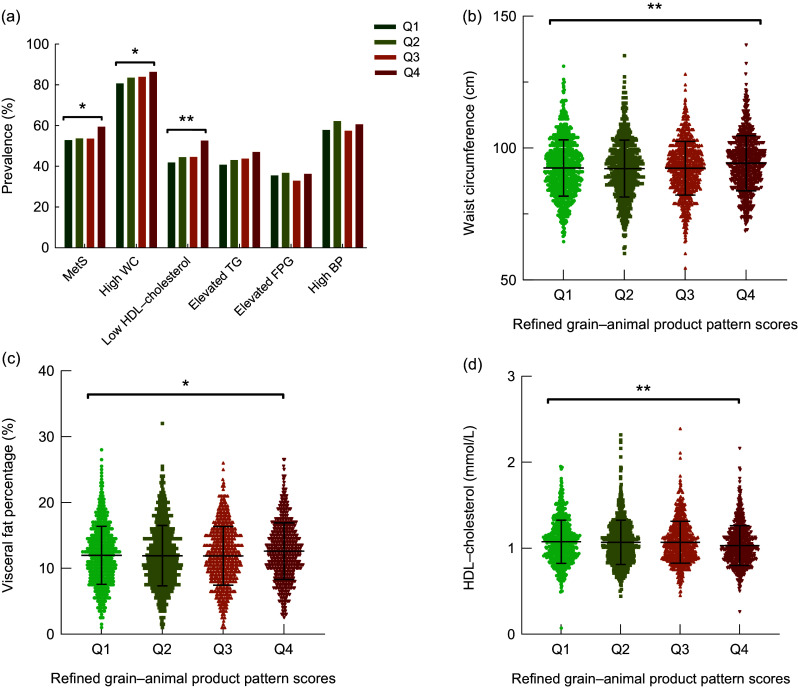
Figure 1 long description. Associations between the refined grain–animal product dietary pattern and metabolic syndrome and its components. (a) Prevalence of MetS and its individual components across quartiles (Q1–Q4) of the refined grain–animal product pattern score. (b) Waist circumference, (c) visceral fat percentage and (d) HDL-cholesterol levels according to dietary pattern quartiles. MetS, metabolic syndrome; WC, waist circumference; FPG, fasting plasma glucose; BP, blood pressure. *P < 0·05; **P < 0·001.

A total of 3208 subjects were included in the study, with a mean age of 53·1 (sd 10·8) years, of whom 1576 (49·1 %) were male. 1762 (54·9 %) individuals met the diagnostic criteria for MetS. (Figure 1) The prevalence of high WC, high blood pressure, low HDL-cholesterol, hypertriglyceridaemia and elevated FPG was 83·6 %, 59·5 %, 45·9 %, 43·7 % and 35·4 %, respectively (see online supplementary material, Supplemental Table 2). Men have a higher prevalence of MetS, low HDL-cholesterol and elevated FPG than women (58·2 % *v*. 51·8 %, 55·9 % *v*. 36·3 %, 37·6 % *v*. 33·2 %, all *P* < 0·05), whereas women had a higher prevalence of abdominal obesity than men (87·3 % *v*. 79·8 %, *P* < 0·001) (see online supplementary material, Supplemental Figure 1). Age-specific MetS prevalence increased with age in both men and women, peaking in the 50–60-year age group. Among 3208 participants, 94·9 % exhibited at least one abnormal metabolic component. The prevalence of individuals with one to five abnormal components was 15·1 %, 24·8 %, 25·7 %, 19·9 % and 9·4 %, respectively.

Compared with the control group, the MetS group was older (53·6 (sd 10·3) *v*. 52·5 (sd 11·4), *P* = 0·003), had a greater proportion of males (52·0 % *v*. 46·5 %, *P* < 0·001) and exhibited a higher prevalence of abdominal obesity (85·3 % *v*. 50·5 %, *P* < 0·001). (Table 1) Regarding anthropometric measurements and cardiovascular metabolic-related laboratory indices, individuals in the MetS group generally had higher levels of those indices, except for HDL-cholesterol, which was lower compared with the control group (*P* < 0·001). In terms of lifestyle factors, a greater proportion of participants in the MetS group reported low levels of physical activity (*P* < 0·001) and extended sedentary durations compared with the control group (*P* = 0·030).

**Table 1.** Characteristics of study population and comparison between metabolic syndrome and controls Table 1 long description.

Characteristics, *n* (%)	Total		MetS		Control		*P*-value
	*n*	%	*n*	%	*n*	%	
*n*	3208		1762	54·9	1446	45·1	*–*
Age (yeas)							
Median	53·1		53·6		52·5		0·003
sd	10·8		10·3		11·4		
Male	1576	49·1	917	52·0	659	45·6	< 0·001
Married/cohabiting	2698	84·1	1498	85·0	1200	83·0	0·118
Primary education	2012	62·7	1114	63·2	898	62·1	0·513
Smoking							
Never	2680	83·5	1444	82·0	1236	85·5	0·002
Former	391	12·2	224	12·7	167	11·5	
Current smoking	137	4·3	94	5·3	43	3·0	
Drinking	132	4·1	80	4·5	52	3·6	0·180
	Mean	sd	Mean	sd	Mean	sd	
Waist circumference (cm)	92·8	10·6	97·0	9·2	87·7	9·9	< 0·001
Male	92·9	10·0	97·1	8·5	86·7	8·6	< 0·001
Female	92·7	9·8	96·6	9·8	88·6	10·7	< 0·001
Abdominal obesity							
* n*	2233		1503		730		< 0·001
%	69·6		85·3		50·5		
BMI (kg/m^2^)	27·5	4·7	29·1	4·3	25·6	4·4	< 0·001
	*n*	%	*n*	%	*n*	%	
Underweight (< 18·5)	25	0·8	2	0·1	24	1·6	< 0·001
Normal (18·5∼)	701	22·0	165	9·4	536	37·2	
Overweight (24·0∼)	1078	33·7	585	33·3	493	34·1	
Obesity (28·0∼)	1395	43·5	1004	57·2	391	27·0	
	Mean	sd	Mean	sd	Mean	sd	
Visceral fat percentage (%)	12·1	4·4	10·3	4·0	13·6	4·2	< 0·001
SBP (mmHg)	132·8	23·0	138·5	22·8	125·9	21·2	< 0·001
DBP (mmHg)	82·7	14·0	85·9	14·0	78·8	13·2	< 0·001
FPG (mmol/l)	5·8	1·9	6·3	2·3	5·2	1·0	< 0·001
LDL-cholesterol (mmol/l)	3·02	0·75	3·11	0·78	2·91	0·72	< 0·001
HDL-C (mmol/l)	1·06	0·24	0·96	0·21	1·19	0·23	< 0·001
Triglycerides (mmol/l)							
Median	1·48		1·97		1·12		< 0·001
IQR	1·04–2·13		1·43–2·72		0·86–1·43		
	*n*	%	*n*	%	*n*	%	
Physical activity							
High-level	1332	41·5	677	38·4	655	45·3	< 0·001
Medium-level	1364	42·5	796	45·2	568	39·3	
Low-level	512	16·0	289	16·4	223	15·4	
Sedentary time							
< 3 h	2053	64·8	1093	62·9	960	67·2	0·030
3～6 h	798	25·2	468	27·0	330	23·1	
≥ 6 h	317	10·0	178	10·1	139	9·7	
PSQI							
Median	4		4		4		0·620
IQR	2–7		2–7		2–7		

Data were shown as *n* (%), mean (sd) or median (interquartile), as appropriate. *P* values were calculated using *t* test for normally distributed variables, the Mann–Whitney *U* for skewed variables and the *χ* ^2^ test for categorical variables. Mets, metabolic syndrome; SBP, systolic blood pressure; DBP, diastolic blood pressure; FPG, fasting plasma glucose; PSQI, Pittsburgh sleep quality index.

### Dietary patterns

Principal component analysis identified four major dietary patterns (eigenvalues ≥ 1·0) accounting for 54·9 % of total variance across thirteen food groups, with individual contributions of 23·3 % (eigenvalue = 3·03), 13·8 % (1·80), 10·0 % (1·30) and 7·9 % (1·03), as visualised in the scree plot (see online supplementary material, Supplemental Figure 2). The first dietary pattern, referred to as the refined grain–animal product pattern, was characterised by high intake of flour-based food (factor loading: 0·83), fruits (0·75) and livestock meat and products (0·59). The second pattern, termed the protein-dessert-nut-enriched pattern, was characterised by high intake of aquatic products (0·70), poultry and products (0·66), desserts (0·65) and nuts (0·60). The third pattern, referred to as the vegetable–egg dietary pattern, was characterised by high consumption of vegetables (0·89) and eggs (0·79). The fourth pattern, termed the whole grain and legume-based pattern, was defined by high intake of coarse grains (0·79) and legumes and legume products (0·54) (see online supplementary material, Supplemental Table 3).

The prevalence of MetS and its individual components varied significantly across quartiles of the refined grain–animal product dietary pattern score (Figure 1(a)). Participants in the highest quartile (Q4) exhibited a markedly higher prevalence of MetS compared with those in the lowest quartile (Q1) (*P* < 0·05). Similarly, the prevalence of elevated WC, visceral fat and reduced HDL-cholesterol levels increased progressively from Q1 to Q4. Quantitative analysis showed that WC and visceral fat percentage (Figure 1(b) and (c)) were significantly higher in Q4, while HDL-cholesterol levels (Figure 1(d)) were significantly lower, indicating a worsening metabolic profile with higher adherence to this dietary pattern. In addition, individuals in higher quartiles of this dietary pattern were more likely to be current smokers and alcohol consumers (*P* < 0·05). They also exhibited higher levels of physical activity, yet reported a higher prevalence of poor sleep quality (Pittsburgh sleep quality index ≥ 7), which was significantly elevated across quartiles (*P* < 0·001). (Table 2) However, no significant associations were found between MetS and the other three dietary patterns identified.

**Table 2.** Characteristics of study participants by quartiles of refined grain–animal product dietary pattern Table 2 long description.

Characteristics, *n* (%)	Q1		Q2		Q3		Q4		*P*-value
	*n*	%	*n*	%	*n*	%	*n*	%	
*n*	802		802		802		802		
Age (years)									
Mean	53·1		54·0		53·3		52·0		0·002
sd	10·7		11·5		11·1		9·8		
Male	398	49·6	371	46·3	362	45·1	445	55·5	< 0·001
Married/cohabiting	674	84·0	685	85·4	660	82·3	679	84·7	0·365
Primary education	524	65·3	506	63·1	516	64·3	466	58·1	0·033
Smoking									
Never	683	85·2	681	84·9	692	86·3	624	77·8	< 0·001
Former	26	3·2	37	4·6	28	3·5	46	5·7	
Current smoking	93	11·6	84	10·5	82	10·2	132	16·5	
Drinking	29	3·6	38	4·7	18	2·2	47	5·9	0·002
	Mean	sd	Mean	sd	Mean	sd	Mean	sd	
Waist circumference (cm)	92·4	10·6	92·2	10·8	92·4	10·2	94·3	10·5	< 0·001
Male	91·9	10·3	92·8	10·5	92·6	8·8	94·0	10·2	0·022
Female	92·9	10·9	91·7	11·0	92·1	11·2	94·5	10·8	0·002
Abdominal obesity									
*n*	536		550		565		582		0·075
%	66·8		68·6		70·5		72·6		
BMI, (kg/m^2^)									
Mean	27·4		27·3		27·3		28·1		0·001
sd	4·7		4·6		4·6		4·7		
	*n*	%	*n*	%	*n*	%	*n*	%	
Underweight (< 18·5), *n* (%)	8	1·0 %	10	1·2 %	7	0·9 %	1	0·1 %	0·010
Normal (18·5∼), *n* (%)	194	24·3 %	172	21·5 %	186	23·2 %	149	18·7 %	
Overweight (24·0∼), *n* (%)	261	32·7 %	289	36·1 %	268	33·4 %	260	32·6 %	
Obesity (28·0∼), *n* (%)	336	42·1 %	330	41·2 %	341	42·5 %	388	48·6 %	
	Mean	sd	Mean	sd	Mean	sd	Mean	sd	
Visceral fat percentage (%)	12·0	4·4	12·0	4·6	11·9	4·4	12·6	4·3	0·002
SBP (mmHg)	132·2	21·8	134·5	23·9	131·6	22·5	133·1	23·5	0·068
DBP (mmHg)	82·5	13·7	83·3	14·4	82·2	13·9	82·6	14·1	0·405
LDL-cholesterol (mmol/l)	3·04	0·77	3·02	0·75	3·04	0·75	3·00	0·76	0·698
HDL-cholesterol (mmol/l)	1·08	0·25	1·07	0·25	1·07	0·24	1·03	0·23	0·001
TAG (mmol/l)									
Median	1·48		1·48		1·46		1·52		0·164
IQR	1·04–2·08		1·03–2·13		1·04–2·11		1·05–2·30		
FPG (mmol/l)	5·7	1·7	5·8	1·8	5·7	1·9	5·8	2·0	0·586
	*n*	%	*n*	%	*n*	%	*n*	%	
Physical activity									
High level	212	26·4	302	37·7	322	40·1	496	61·9	< 0·001
Medium level	365	45·5	370	46·1	412	51·4	217	27·0	
Low level	225	28·1	130	16·2	68	8·5	89	11·1	
Sedentary time									
< 3 h	370	47·3	534	67·5	584	73·1	565	71·1	< 0·001
3–6 h	207	26·4	180	22·8	199	24·9	212	26·7	
> = 6 h	206	26·3	77	9·7	16	2·0	18	2·2	
PSQI > = 7	150	18·7	126	15·7	167	20·8	198	24·7	< 0·001
MetS and component									
MetS	424	52·9	431	53·7	430	53·6	477	59·5	0·028
High WC	647	80·7	670	83·5	673	83·9	693	86·4	0·021
High BP	464	57·9	499	62·2	461	57·5	486	60·6	0·163
Low HDL-cholesterol	336	41·9	357	44·5	358	44·6	422	52·6	< 0·001
Elevated TAG	327	40·8	346	43·1	351	43·8	377	47·0	0·091
Elevated FPG	285	35·5	295	36·8	264	32·9	291	35·4	0·375

Participants were grouped into quartiles based on dietary pattern scores. SBP, systolic blood pressure; DBP, diastolic blood pressure; FPG, fasting plasma glucose; PSQI, Pittsburgh sleep quality index; Mets, metabolic syndrome; WC, waist circumference; BP, blood pressure.

### Association between the refined grain–animal product pattern and metabolic syndrome

**Figure 2.**
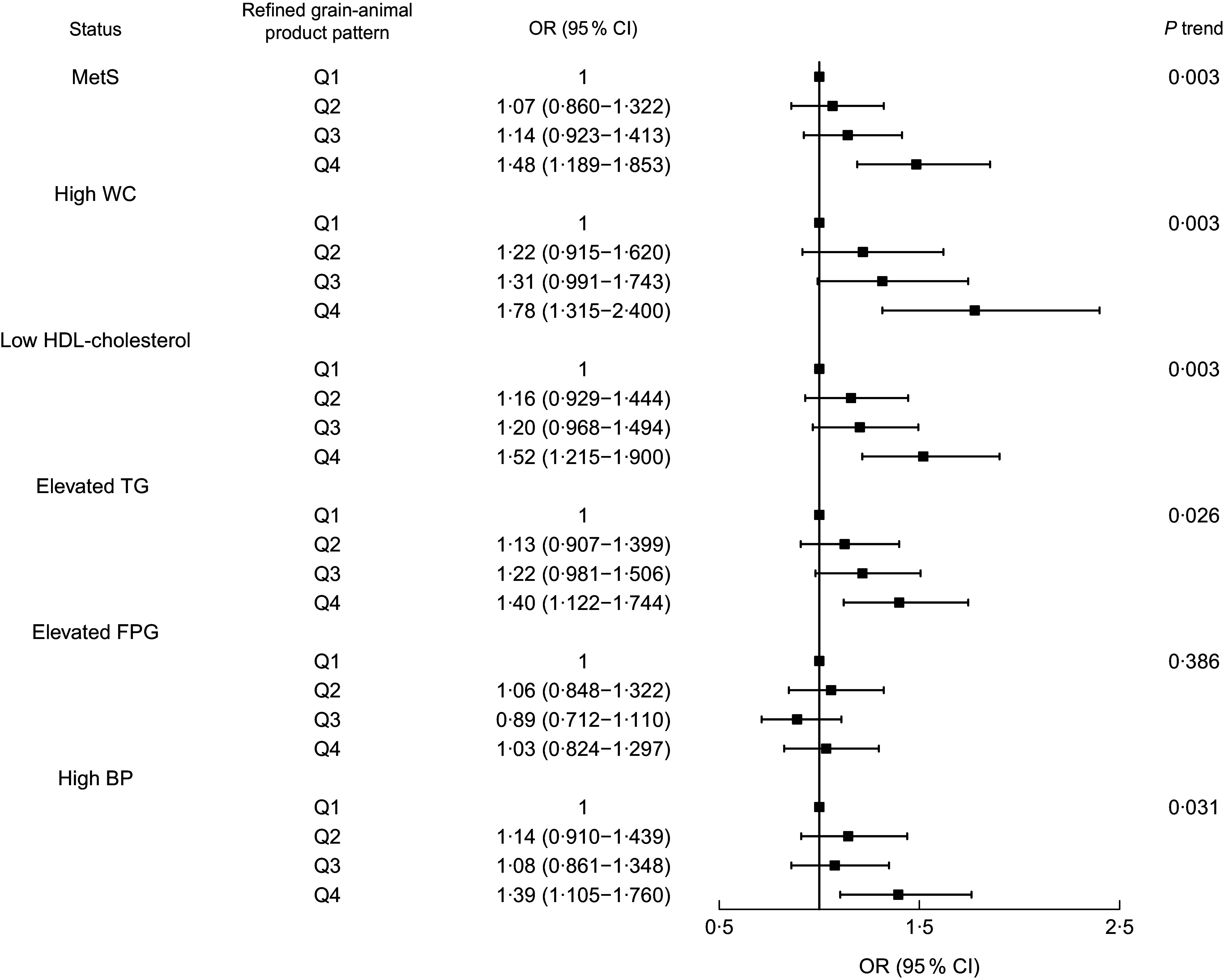
Figure 2 long description. Forest plot of multivariable logistic regression results for metabolic syndrome, its components and refined grain–animal product pattern. Participants were categorised into quartiles based on dietary pattern scores, with the lowest quartile (Q1) serving as the reference group. Multivariable models were adjusted for age, sex, education level, smoking status, alcohol consumption, physical activity, sedentary time and sleep quality. *P* value for trend was calculated by Mantel–Haenszel *χ* ^2^ test. MetS, metabolic syndrome; FPG: fasting plasma glucose.

**Figure 3.**
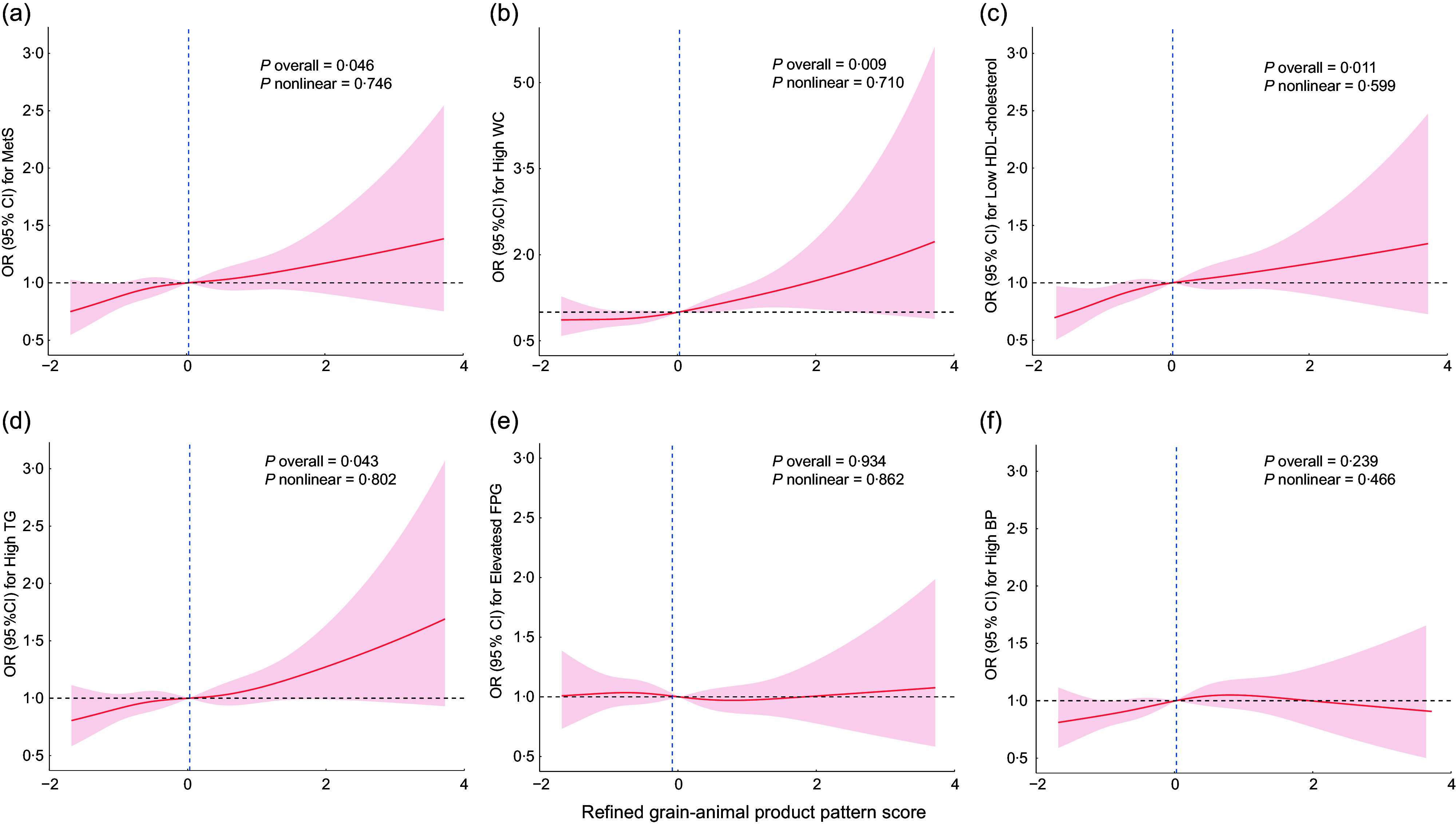
Independent dose–response association between refined grain–animal product dietary pattern scores and metabolic syndrome and its individual components. (a) Metabolic syndrome; (b) high waist circumference; (c) low HDL-cholesterol; (d) high TAG; (e) elevated fasting plasma glucose and (f) high blood pressure. Restricted cubic splines were constructed with three knots located at the 5th, 50th and 95th percentiles of the dietary pattern score distribution. Multivariable models were adjusted for age, sex, higher education, marital status, smoking, drinking, sedentary time and sleep quality (PSQI score). MetS, metabolic syndrome; WC, waist circumference; FPG, fasting plasma glucose; BP, blood pressure.

In a multiple logistic regression analysis, higher scores in the refined grain–animal product dietary pattern were associated with an increased likelihood of MetS and multiple components. After adjusting for potential confounders, a significant linear trend was observed in the odds of MetS across increasing quartiles of the refined grain–animal product pattern (*P*-trend = 0·003), with ORs of 1·07 (95 % CI: 0·860, 1·322), 1·14 (95 % CI: 0·923, 1·413) and 1·48 (95 % CI: 1·189, 1·853) for Q2 to Q4, respectively, compared to the lowest quartile (Q1). (Figure 2). The increasing trend remained significant for individual components, including high WC, hypertriglyceridemia, low HDL-cholesterol, and high BP (*P*-trend < 0·05). No significant associations were found for high BP and elevated FPG. Additionally, the other three dietary patterns showed no statistically significant associations with MetS or its individual components. To further explore the dose–response relationship, restricted cubic spline models were constructed. As shown in Figure 3, higher scores in the refined grain–animal product pattern were associated with an increased risk of MetS in a nonlinear, dose-dependent manner (*P*-overall = 0·046). Similar dose–response relationships were observed for low HDL-cholesterol, hypertriglyceridaemia and high WC (*P*-overall < 0·05), reinforcing the positive association between this dietary pattern and key metabolic abnormalities.

Mediation analysis revealed that the refined grain–animal product dietary pattern was significantly associated with low HDL-cholesterol (total effect = 0·032, *P* = 0·032), with visceral fat percentage acting as a partial mediator (see online supplementary material, Supplemental Figure 3). The indirect effect through visceral fat accounted for 17·2 % of the total effect (effect = 0·005, 95 % CI: 0·001, 0·010, *P* = 0·006), while the direct effect remained significant (effect = 0·027, 95 % CI: 0·012, 0·040, *P* = 0·026). These results suggest that visceral fat partially mediates the link between this dietary pattern and low HDL-cholesterol. In contrast, mediation analyses for other MetS components and adiposity indicators (e.g. BMI, WC) showed no significant indirect effects.

### Sensitivity analyses

In the sensitivity analysis, three alternative diagnostic criteria for MetS and its components were employed to investigate the association with refined grain–animal product pattern. These criteria included NCEP-ATP III definitions, as modified in 2004; IDF definitions from 2005; and the JIS definitions from 2009 (see online supplementary material, Supplemental Figures S4–S6). The dose–response relationship between refined grain–animal product pattern scores and MetS and its components were also analysed using the three different diagnostic criteria (see online supplementary material, Supplemental Figure S7). The findings demonstrated consistency across the three distinct diagnostic criteria, all of which indicated an association between elevated scores in the refined grain–animal product pattern and the presence of MetS or its individual components.

## Discussion

This large-scale population-based study in western China focused on the association between MetS and dietary patterns, emphasising the importance of region-specific dietary behaviours in shaping metabolic health. Our study uncovered the following novel findings. First, the overall prevalence of MetS in our study was 54·9 %, with considerable variation across demographic groups. Particularly, abdominal obesity was more prevalent in women than in men. Second, four distinct local dietary patterns were identified through principal component analysis. The dominant pattern, characterised by refined grains–animal products, was associated with an increased risk of MetS, abdominal obesity and dyslipidaemia regardless of the diagnostic criteria for MetS. Finally, the refined grain–animal product dietary pattern was significantly associated with low HDL-cholesterol, with visceral fat percentage acting as a partial mediator, suggesting a potential pathway linking dietary habits to lipid abnormalities. Our study underscores the need for lifestyle modifications and targeted interventions to manage MetS effectively, particularly in regions with high consumption of refined grains and animal products. Targeted dietary interventions that promote balanced nutrition and reduce the intake of refined grains and animal-based foods may help mitigate the risk of MetS and its associated components, especially in populations with regional dietary patterns closely linked to metabolic disturbances.

MetS presents an escalating public health burden, with prevalence varying across populations due to differences in diagnostic criteria, demographics and lifestyle factors^(1)^. Based on data from the PCCDX cohort, our study identified a MetS prevalence of 54·9 % in western China, predominantly driven by obesity, hypertension and low HDL-cholesterol levels. This rate is comparable to the 53·4 % reported in the UK Biobank study^(24)^, which included over 300 000 participants with a mean age of 56·4 years, similar to that in our study population. In contrast, the recent China Multi-Ethnic Cohort (CMEC) reported a considerably lower prevalence of 19·4 %^(25)^. This discrepancy may be explained by differences in diagnostic criteria and the younger age profile of China Multi-Ethnic Cohort participants. Notably, the China Multi-Ethnic Cohort cohort does not include populations from western China. The high prevalence observed in western China may be attributed to the substantial burden of metabolic risk factors^(16,17,26)^, including abdominal obesity, hypertension, dyslipidaemia, elevated blood glucose levels, as well as distinctive lifestyle, dietary patterns^(12)^ and environmental exposures such as air pollution^(27)^. Notably, central obesity was the most prominent feature, with abdominal obesity accounting for over 80 % of MetS cases. These patterns suggest a pronounced imbalance in energy metabolism and adipose tissue distribution, which are closely linked to insulin resistance, inflammation, and altered lipid metabolism^(28,29)^. Particularly, abdominal obesity was more prevalent in women than in men in our study. This observed sex-related disparity is likely attributable to postmenopausal hormonal shifts, leading to central adiposity due to declining ovarian function^(30)^. Additionally, increasing trends of physical inactivity and prolonged sedentary behavior may contribute to the growing burden of central obesity among women^(31)^. These insights highlight the importance of context-specific approaches to understanding and managing MetS, particularly in populations with distinct socio-demographic and environmental profiles. However, such heterogeneity is often obscured by standardised definitions that may inadequately capture regionally salient risks. By incorporating data from this underrepresented region, our study provides valuable evidence to refine the national narrative on metabolic health in China.

Our study used principal component analysis to identify a refined grain–animal product dietary pattern characterised by high sugar and fat intake among the population in western China, which was significantly associated with Mets, abdominal obesity and dyslipidaemia. Compared with the traditional Chinese diet (primarily grains and vegetables)^(32)^ and the modern diet (including more dairy and processed foods)^(33)^ commonly found in Eastern and Central China, the refined grain–animal product pattern combines high animal protein with high-glycemic-index carbohydrates and is notably low in dietary fiber, reflecting a distinct regional nutritional structure to western China. The restricted cubic spline analysis revealed a nonlinear dose–response relationship between the refined grain–animal product pattern and metabolic abnormalities. The risk of MetS and its components increased gradually at lower dietary pattern scores but rose sharply beyond a certain level, suggesting a potential threshold effect and emphasising that excessive consumption of refined grains and animal products may disproportionately increase metabolic risk. Further analysis revealed a strong association between this dietary pattern and low HDL-cholesterol levels, with visceral fat percentage acting as a partial mediator. This finding suggests that the pattern may contribute to lipid metabolism disruption by promoting visceral fat accumulation. Notably, neither BMI nor WC showed a significant mediating effect, likely because visceral adipose tissue, which is deposited around intra-abdominal organs, is a highly metabolically active pathogenic fat depot that directly drives systemic inflammation, insulin resistance and dyslipidaemia, whereas BMI and WC cannot distinguish between fat and lean mass or specify fat distribution^(34,35)^. Previous studies have confirmed that visceral fat can secrete inflammatory cytokines and free fatty acids, which inhibit HDL-cholesterol synthesis and accelerate its clearance, thereby supporting the potential mechanistic pathway of ‘dietary pattern–visceral obesity–lipid dysregulation’^(36,37)^. In contrast, the effects of visceral fat on other metabolic components, such as fasting glucose, blood pressure and TAG, are likely to be more indirect and mediated through multiple regulatory pathways, including insulin signaling, endothelial function and renal sodium handling^(38)^. Therefore, this specific mediating effect was primarily observed for HDL-cholesterol rather than for other metabolic outcomes. Moreover, similar animal-based dietary patterns have been associated with CVD risk in other Asian countries such as Japan^(39)^, but meta-analyses across Asia have not yielded consistent results^(12,40)^, possibly reflecting the heterogeneity in the sources, types and combinations of animal protein and their health impacts across different cultural contexts. Given that the dietary habits in Xinjiang, characterised by animal-based consumption patterns, are more aligned with those of Central Asian countries than with traditional Chinese diets, this phenomenon further underscores the need for region-specific dietary and health research^(12)^. In conclusion, this study fills the gap in the research on the relationship between dietary behaviour and metabolic health in western China, revealing a potential pathway through which a specific dietary structure may contribute to metabolic abnormalities by affecting body fat distribution, providing empirical evidence for the development of nutrition interventions and public health strategies based on regional cultural characteristics.

Our study has several limitations that should be acknowledged. First, dietary intake was assessed using semi-quantitative FFQ, a method inherently limited by recall bias, misreporting and reliance on the accuracy of food composition databases, which may not comprehensively capture regional dietary variations. Second, a convenience sampling approach was adopted, which may have introduced sampling bias. Participants were recruited from a single town predominantly inhabited by Uyghur populations, and thus the findings may not be fully generalisable to other ethnic or regional groups in China. Third, the identified dietary patterns may reflect region-specific cultural practices and thus may not be generalisable beyond the study population in western China. However, the methodological framework remains applicable for examining culturally distinct dietary behaviours in other contexts. Finally, due to the impracticality and ethical constraints of assigning individuals to long-term dietary exposures, the study employed an observational design, as is standard in nutritional epidemiology. Consequently, causal inferences cannot be established, and the potential for residual confounding and reverse causality cannot be excluded.

### Conclusions

This large-scale, population-based study in western China reveals a high prevalence of MetS, with region-specific dietary patterns playing a critical role in its development. Notably, a refined grain–animal product dietary pattern was significantly associated with MetS, abdominal obesity, high BP and dyslipidaemia. Visceral fat percentage was identified as a potential mediator in this association, highlighting a plausible pathway through which dietary behaviours may influence metabolic health. These findings underscore the importance of culturally and regionally tailored dietary interventions to address the growing burden of MetS, especially in underrepresented populations with unique nutritional structures.

## Supporting information

10.1017/S1368980026102316.sm001 Zhao et al. supplementary material Zhao et al. supplementary material

## Data Availability

The data and material supporting the findings of the study are available from the corresponding authors upon reasonable request.

## Supplementary material

For supplementary material accompanying this paper visit https://doi.org/10.1017/S1368980026102316

## Figure 1 Long description

Panel A: A vertical bar graph shows the prevalence of metabolic syndrome (MetS) and its individual components across quartiles (Q1 to Q4) of the refined grain-animal product pattern score. The x-axis represents different components of MetS, including MetS, high waist circumference (WC), low HDL-cholesterol, elevated triglycerides (TG), elevated fasting plasma glucose (FPG), and high blood pressure (BP). The y-axis represents the prevalence percentage. The bars are color-coded by quartiles, with Q1 in green, Q2 in light green, Q3 in orange, and Q4 in red. Significant differences are marked with asterisks, indicating statistical significance. Panel B: A scatter plot displays waist circumference in centimeters across quartiles of the refined grain-animal product pattern scores. The x-axis represents the quartiles (Q1 to Q4), and the y-axis represents waist circumference in centimeters. Each dot represents an individual data point, with the quartiles color-coded as in Panel A. Panel C: A scatter plot shows visceral fat percentage across quartiles of the refined grain-animal product pattern scores. The x-axis represents the quartiles (Q1 to Q4), and the y-axis represents visceral fat percentage. Each dot represents an individual data point, with the quartiles color-coded as in Panel A. Panel D: A scatter plot depicts HDL-cholesterol levels in mmol/L across quartiles of the refined grain-animal product pattern scores. The x-axis represents the quartiles (Q1 to Q4), and the y-axis represents HDL-cholesterol levels in mmol/L. Each dot represents an individual data point, with the quartiles color-coded as in Panel A. Significant differences are marked with asterisks, indicating statistical significance.

Navigate back to Figure 1.

## Figure 2 Long description

A table with six rows and five columns comparing the odds ratios (OR) and 95 percent confidence intervals (CI) for different metabolic syndrome components across quartiles (Q1 to Q4) of a refined grain-animal product pattern. The components include MetS, High WC, Low HDL-cholesterol, Elevated TG, Elevated FPG, and High BP. Each row represents a different component, and the columns show the OR (95 percent CI) for Q1 to Q4, with Q1 as the reference group. The P trend value for each component is also listed. Notable trends include increasing ORs for MetS, High WC, Low HDL-cholesterol, Elevated TG, and High BP from Q1 to Q4, indicating a positive association with higher quartiles of the refined grain-animal product pattern. Elevated FPG shows no significant trend across quartiles.

Navigate back to Figure 2.

## Table 1 Long description

The table compares characteristics of a study population between metabolic syndrome (MetS) and control groups. It has 32 rows and 10 columns. The columns are labeled Total, MetS, and Control, each with sub-columns for count (n) and percentage (%). The rows include characteristics such as age, gender, marital status, education, smoking status, drinking status, waist circumference, abdominal obesity, body mass index (BMI), visceral fat percentage, systolic blood pressure (SBP), diastolic blood pressure (DBP), fasting plasma glucose (FPG), low-density lipoprotein cholesterol (LDL-C), high-density lipoprotein cholesterol (HDL-C), triglycerides, physical activity level, sedentary time, and Pittsburgh Sleep Quality Index (PSQI). Each row provides data for the total population, MetS group, and control group, along with P-values indicating statistical significance. Notable trends include higher median age, greater proportion of males, higher prevalence of abdominal obesity, higher BMI, higher visceral fat percentage, higher blood pressure, higher FPG, higher LDL-C, lower HDL-C, higher triglycerides, lower physical activity levels, and longer sedentary time in the MetS group compared to the control group.

Navigate back to Table 1.

## Table 2 Long description

The table presents characteristics of study participants across quartiles of a dietary pattern, with data on age, gender, marital status, education, smoking, drinking, waist circumference, abdominal obesity, BMI, blood pressure, cholesterol levels, fasting plasma glucose, physical activity, sedentary time, and metabolic syndrome components. The table has 30 rows and 13 columns, including headers and quartile divisions. Each row provides specific data points for each quartile, with percentages and means where applicable. Notable trends include variations in waist circumference, BMI, and the prevalence of metabolic syndrome components across quartiles.

Navigate back to Table 2.
