# Supplementary material for: Dietary patterns in Xinjiang, Western China and associations with metabolic syndrome: a population-based study
Source: Public Health Nutr. 2026 Apr 1;29(1):e139. doi: 10.1017/S1368980026102316 (PMC13434563; doi:10.1017/S1368980026102316)
Supplement: Supplementary file 1 [file S1368980026102316sup001.docx]

***Supplementary materials***

**Dietary Patterns in Western China and Associations with Metabolic Syndrome: A Population-Based Study**

*Qian Zhao, Juan Wang, Xier Zulipiye, Na Yang,* *Jun Liu, Aboduhapaer Tuersunjiang, Hui Peng, Yining Yang*

**Method:**

**Alternative Diagnostic Criteria for MetS**

*NCEP-ATP III (modified in 2004)* *Definitions*

MetS was defined according to the American Heart Association/National Heart, Lung, and Blood Institute Scientific Statement, which updated the National Cholesterol Education Program Adult Treatment Panel III (NCEP-ATP III) criteria in 2005 ^1^, as having three or more of the following factors:

(1) a waist circumference of 102 cm for men and 88 cm for women;

(2) a systolic blood pressure of 130 mmHg or a diastolic blood pressure of 85 mmHg or receiving anti-hypertension treatment;

(3) a fasting triglyceride level of 1.7 mmol/L or receiving corresponding treatment;

(4) a high-density lipoprotein cholesterol (HDL-C) level of <1.03 mmol/L for men and <1.30 mmol/L for women or receiving corresponding treatment;

(5) a fasting plasma glucose (FPG) level of 5.6 mmol/L or receiving anti-diabetes treatment or reporting previously physician-diagnosed diabetes.

In the text, the diagnostic criteria are referred to as NCEP-ATP III (modified in 2004) definitions.

*IDF (2005) Definitions*

MetS was defined according to the International Diabetes Federation (IDF) criteria in 2005^2^, which requires a mandatory obesity component (a waist circumference of 94 cm for men and 80 cm for women) along with at least two of the following four components:

(1) fasting glucose ≥100 mg/dL or receiving hypoglycemic treatment;
(2) triglycerides ≥150 mg/dL or receiving hyperlipemic therapy;
(3) a high-density lipoprotein cholesterol (HDL-C) level of <40 mg/dL for men and <50 mg/dL for women or receiving corresponding treatment;
(4) a systolic blood pressure of 130 mmHg or a diastolic blood pressure of 85 mmHg or receiving anti-hypertension treatment.

In the text, the diagnostic criteria are referred to as IDF (2005) definitions.

*JIS 2009 Definitions*

According to the joint interim statement of the International Diabetes Federation Task Force on Epidemiology and Prevention (JIS) criteria in 2009^3^, we defined MetS for participants with abdominal obesity who met at least two out of the following four criteria: Abdominal obesity (applicable to China): a waist circumference of 85 cm for men and 80 cm for women.

(1) fasting glucose ≥ 5.6 mmol/L or treatment for type 2 diabetes;
(2) HDL cholesterol≥ 1.29 mmol/L in women or drug treatment for lipid abnormality;
(3) triglycerides ≥ 1.7 mmol/L or drug treatment for lipid abnormality;
(4) and systolic BP ≥ 130 mmHg or diastolic BP ≥ 85 mmHg, or drug treatment for hypertension

In the text, the diagnostic criteria are referred to as JIS 2009 definitions.

**Supplementary Table 1.** Food groups categorized by nutritional composition and taxonomy for dietary analysis

|  | **Food group** | **Food items** |
| --- | --- | --- |
| 1 | Rice | Rice |
| 2 | Flour-based food | Noodles/Steamed stuffed buns/Mantou/Naan |
| 3 | Coarse grains | Maize/Foxtail millet/Sweet potato/ Oat |
| 4 | Desserts | Sweets/Pastries/Cakes/Desserts |
| 5 | Livestock meat and products | Beef/Lamb |
| 6 | Poultry and products | Chicken/Duck |
| 7 | Aquatic products | Fish/Shrimp/Crab/Shellfish |
| 8 | Eggs | Eggs/Duck eggs/Quail eggs/Goose eggs |
| 9 | Vegetables | Leafy Green Vegetables/Root Vegetables, etc. |
| 10 | Fruits | Apple/Banana/Strawberry/Watermelon/Orange/Peach/Hami Melon, etc. |
| 11 | Nuts | Peanut/Walnut/Almond/Almond |
| 12 | Legumes and products | Tofu/Soft Tofu (Tofu Pudding)/Dried Tofu/Fried Tofu Puffs |
| 13 | Yogurt | Homemade/Store-Bought Yogurt |

**Supplementary Table 2.** Prevalence of metabolic syndrome and components according to sex (n/%)

|  | MetS | High waist circumference | High blood pressure | Low  HDL-C | Elevated triglycerides | Elevated  FPG |
| --- | --- | --- | --- | --- | --- | --- |
| Total | 1 762 (54.9) | 2 683 (83.6) | 1 910 (59.5) | 1 473 (45.9) | 1 401 (43.7) | 1 135 (35.4) |
| Male | 917 (58.2) | 1 258 (79.8) | 954 (60.5) | 881 (55.9) | 685 (43.5) | 593 (37.6) |
| Female | 845 (51.8) | 1 425 (87.3) | 956 (58.6) | 592 (36.3) | 716 (43.9) | 542 (33.2) |
| *P-*value | <0.001 | <0.001 | 0.259 | <0.001 | 0.816 | 0.009 |

*P*-values were calculated using the Chi-squared test. MetS, metabolic syndrome; HDL-C, high-density lipoprotein cholesterol; FPG, fasting plasma glucose.

**Supplementary Table 3.** Factor loadings of food groups for dietary patterns derived from PCA

| **Food group** | **PCA1** | **PCA2** | **PCA3** | **PCA4** |
| --- | --- | --- | --- | --- |
| Rice | 0.44 | 0.12 | -0.27 | 0.34 |
| Flour-based food | 0.83 | -0.01 | -0.18 | 0.01 |
| Coarse grains | 0.16 | -0.19 | 0.04 | 0.79 |
| Desserts | -0.11 | 0.65 | -0.05 | 0.11 |
| Livestock meat and products | 0.59 | 0.07 | 0.27 | 0.30 |
| Poultry and products | 0.08 | 0.66 | -0.07 | 0.04 |
| Aquatic products | 0.15 | 0.70 | -0.06 | -0.29 |
| Eggs | -0.24 | 0.08 | 0.79 | 0.01 |
| Vegetables | 0.17 | -0.07 | 0.89 | 0.03 |
| Fruits | 0.75 | 0.10 | 0.16 | -0.09 |
| Nuts | 0.15 | 0.60 | 0.26 | -0.05 |
| Legumes and products | -0.33 | 0.33 | 0.01 | 0.54 |
| Yogurt | -0.05 | 0.03 | -0.07 | 0.04 |

PCA: principal component analysis.

**Supplementary Figure 1.** Prevalence of metabolic syndrome and its components stratified by sex (A), age groups (B, C), and metabolic components (D) in western China.

MetS, metabolic syndrome; WC, waist circumference; BP, blood pressure; TG, triglycerides; HDL-C, high-density lipoprotein cholesterol; FPG, fasting plasma glucose. Lines represent Total (solid orange line), Male (dotted blue line), and Female (dashed yellow line) participants. *P* values denote statistical significance of sex-specific differences for each MetS component (High WC, Elevated TG, Low HDL-C, High BP, Elevated FPG).

**Supplementary Figure 2.** Scree plot illustrating the eigenvalues for each principal component, indicating the amount of total variance explained.

PCA: principal component analysis.

**Supplementary Figure 3.** Mediation analysis of visceral fat in the associations between animal meat and staple food patterns with low HDL-C

HDL-C, high-density lipoprotein cholesterol

**Supplementary Figure 4.** Sensitivity analysis of metabolic syndrome prevalence and its components, along with refined grain-animal product dietary pattern, using NCEP-ATP III criteria. Prevalence of metabolic syndrome and its components stratified by sex (A), age groups (B, C), and the refined grain-animal product dietary pattern and multi-factor logistic regression analysis (D) in western China, based on NCEP-ATP III definitions.

MetS: Metabolic syndrome; TG, triglycerides; HDL-C, high-density lipoprotein cholesterol; NCEP-ATP III: National Cholesterol Education Program Adult Treatment Panel III. Lines represent Total (solid orange line), Male (dotted blue line), and Female (dashed yellow line) participants. *P* values denote statistical significance of sex-specific differences for each MetS component (High WC, Elevated TG, Low HDL-C, High BP, Elevated FPG).

**Supplementary Figure 5.** Sensitivity analysis of metabolic syndrome prevalence and its components, along with refined grain-animal product dietary pattern, using IDF criteria. Prevalence of metabolic syndrome and its components stratified by sex (A), age groups (B, C), and the refined grain-animal product dietary pattern and multi-factor logistic regression analysis (D) in western China, based on IDF criteria.

MetS: Metabolic syndrome; TG, triglycerides; HDL-C, high-density lipoprotein cholesterol; IDF: International Diabetes Federation. Lines represent Total (solid orange line), Male (dotted blue line), and Female (dashed yellow line) participants. *P* values denote statistical significance of sex-specific differences for each MetS component (High WC, Elevated TG, Low HDL-C, High BP, Elevated FPG).

**Supplementary Figure 6.** Sensitivity analysis of metabolic syndrome prevalence and its components, along with refined grain-animal product dietary pattern, using JIS criteria. Prevalence of metabolic syndrome and its components stratified by sex (A), age groups (B, C), and the refined grain-animal product dietary pattern and multi-factor logistic regression analysis (D) in western China, based on JIS criteria.

MetS: Metabolic syndrome; TG, triglycerides; HDL-C, high-density lipoprotein cholesterol; JIS: Joint interim statement of the International Diabetes Federation Task Force on Epidemiology and Prevention. Lines represent Total (solid orange line), Male (dotted blue line), and Female (dashed yellow line) participants. *P* values denote statistical significance of sex-specific differences for each MetS component (High WC, Elevated TG, Low HDL-C, High BP, Elevated FPG).

**Supplementary** **Figure7.** Independent dose-response association of refined grain-animal product dietary pattern with metabolic syndrome and its components (based on three diagnostic criteria). A: NCEP-ATP III (modified in 2004) definitions; B: IDF (2005) Definitions; C: JIS 2009 definitions. Restricted cubic splines were constructed with three knots located at the 5^th^, 50^th^, and 95^th^ percentiles of each exposure.

**Reference**

1. Grundy SM, Cleeman JI, Daniels SR, et al. Diagnosis and management of the metabolic syndrome: an American Heart Association/National Heart, Lung, and Blood Institute Scientific Statement. *Circulation* 2005;112(17):2735-52. doi: 10.1161/CIRCULATIONAHA.105.169404 [published Online First: 2005/09/15]

2. Alberti KG, Zimmet P, Shaw J, et al. The metabolic syndrome--a new worldwide definition. *Lancet* 2005;366(9491):1059-62. doi: 10.1016/S0140-6736(05)67402-8 [published Online First: 2005/09/27]

3. Alberti KG, Eckel RH, Grundy SM, et al. Harmonizing the metabolic syndrome: a joint interim statement of the International Diabetes Federation Task Force on Epidemiology and Prevention; National Heart, Lung, and Blood Institute; American Heart Association; World Heart Federation; International Atherosclerosis Society; and International Association for the Study of Obesity. *Circulation* 2009;120(16):1640-5. doi: 10.1161/CIRCULATIONAHA.109.192644 [published Online First: 2009/10/07]
